# Complete Genome Sequences of One Human Respiratory Syncytial Antigenic Group A Virus from China and Its Four Mouse-Adapted Isolates

**DOI:** 10.1128/genomeA.00062-15

**Published:** 2015-03-05

**Authors:** Ke Zhang, Jie He, Cun Li, Michael E. Bose, Kelly J. Henrickson, Jie Zhou, Bo-Jian Zheng

**Affiliations:** a Department of Microbiology, University of Hong Kong, Hong Kong, China; b Department of Parasitology, Guiyang Medical University, Guizhou, China; c Department of Pediatrics, Medical College of Wisconsin, Milwaukee, Wisconsin, USA

## Abstract

In this study, one human respiratory syncytial antigenic group A virus (HRSV-A-GZ08-0) and its four BALB/c mouse-adapted isolates were sequenced and elucidated. Nineteen nucleotides were mutated between HRSV-A-GZ08-0 and the four mouse-adapted isolates.

## GENOME ANNOUNCEMENT

Worldwide, human respiratory syncytial virus (HRSV) is a main cause of acute lower respiratory tract infection (ALRTI), which can be severe and lead to hospitalization for children under 5 years of age and immunocompromised adults (1). HRSV, known as a nonsegmented enveloped negative-strand RNA virus, belongs to the subfamily *Pneumovirinae* of the family *Paramyxoviridae* with antigenic groups A and B. Currently, antigenic group A is further divided into GA1 to GA7, NA1 to NA2, ON1, and SAA1 genotypes, while group B consists of BA1 to BA10, GB1 to GB4, and SAB1 to SAB3 (2). The initiate sites of 3′ leader (Le) and 5′ trailer (Tr) sequences in the HRSV genome are fully conserved (3). With strong promoters enclosed, Le plays a key role in replication, transcription, and encapsidation, whereas Tr focuses more on genome RNA generation via the mediation from viral RNA-dependent RNA polymerase (4, 5). The mutation in Le or Tr may cause significant modification in HRSV biology.

The HRSV strain HRSV-A-GZ08-0 was clinically isolated in Guangzhou Children’s Hospital, Guangzhou, China, in 2008. Due to severe cytopathogenic effects observed *in vitro*, HRSV-A-GZ08-0 was considered as a candidate strain for establishing an HRSV infection mouse model, so this strain was serially passed in BALB/c mice for over 50 passages. Four mouse-adapted clones (HRSV-A-GZ08-10, HRSV-A-GZ08-12, HRSV-A-GZ08-18, and HRSV-A-GZ08-19) were isolated from one mouse because of high titer generation and purified by plaque assay. The full-length sequencing data of these five HRSV-A isolates were acquired by custom-designed primers, RNA extraction, reverse transcription PCR, and reading by 3730 DNA Analyzer (Thermo, Fisher Scientific, Waltham, MA, USA). The DNA 7500 kit and 2100 Bioanalyzer (Agilent Technologies, Santa Clara, CA, USA) were used as quality controls for concentration of amplicon. Sequence assembly and consensus calling were performed using SeqMan Pro of the Lasergene 11 program suite (DNASTAR, Madison, WI, USA) (6). The nucleotide and protein annotations were presented with GenBank reference KC731483 and CLC Genomic Workbench version 7.0 (Qiagen, Hilden, Germany).

The five newly sequenced HRSVs possessed genomes from 15,193 bp to 15,201 bp, including Le and partial Tr regions. Eleven open reading frames were deduced from genomes for expression of NS1, NS2, N, P, M, SH, G, F, M2-1, M2-2, and L proteins. The four mouse-adapted isolates had nearly identical nucleotide information. Compared with HRSV-A-GZ08-0, they all possessed 5, 4, 3, and 2 nucleotide mutations in G, F, L, and NS1 genes, while N, P, and M genes exhibited 1 mutation, respectively. Notably, 1 mutation (A42G) was found in the Le region of all four mouse isolates and another mutation (A37G) occurred only in HRSV-A-GZ08-19. The nucleotide sequence identities of HRSV-A-GZ08-0 to GenBank whole-genome information of HRSV-A were from 96.97% to 97.30% (7, 8). The phylogenetic analysis showed that HRSV-A-GZ08-0 was assigned as GA2 genotype.

The genome information of HRSV-A-GZ08-0 and its four mouse-adapted isolates would provide valuable information for HRSV mice model research.

### Nucleotide sequence accession numbers.

The complete sequences of HRSV-A-GZ08-0, HRSV-A-GZ08-10, HRSV-A-GZ08-12, HRSV-A-GZ08-18, and HRSV-A-GZ08-19 were deposited in GenBank under the accession numbers KP218910, KP119745, KP119746, KP119747, and KP119748, respectively.
